# Musculoskeletal manifestations in children with Behçet’s syndrome: data from the AIDA Network Behçet’s Syndrome Registry

**DOI:** 10.1007/s11739-023-03215-w

**Published:** 2023-03-07

**Authors:** Carla Gaggiano, Anna Maselli, Petros P. Sfikakis, Katerina Laskari, Gaafar Ragab, Mohamed Tharwat Hegazy, Ahmed Hatem Laymouna, Giuseppe Lopalco, Ibrahim A. Almaghlouth, Kazi Nur Asfina, Ohoud Alahmed, Henrique Ayres Giardini Mayrink, Isabele Parente de Brito Antonelli, Marco Cattalini, Matteo Piga, Jurgen Sota, Stefano Gentileschi, Maria Cristina Maggio, Daniela Opris-Belinski, Gülen Hatemi, Antonella Insalaco, Alma Nunzia Olivieri, Abdurrahman Tufan, Hazan Karadeniz, Riza Can Kardaş, Francesco La Torre, Fabio Cardinale, Achille Marino, Silvana Guerriero, Piero Ruscitti, Maria Tarsia, Antonio Vitale, Valeria Caggiano, Salvatore Telesca, Florenzo Iannone, Veronica Parretti, Micol Frassi, Emma Aragona, Francesco Ciccia, Ewa Wiesik-Szewczyk, Ruxandra Ionescu, Ali Şahin, Nurullah Akkoç, Andrea Hinojosa-Azaola, Samar Tharwat, José Hernández-Rodríguez, Gerard Espinosa, Giovanni Conti, Emanuela Del Giudice, Marcello Govoni, Giacomo Emmi, Claudia Fabiani, Alberto Balistreri, Bruno Frediani, Donato Rigante, Luca Cantarini, the AIDA Network

**Affiliations:** 1 Department of Medical Sciences, Surgery and Neurosciences, Rheumatology Unit, University of Siena and Azienda Ospedaliero-Universitaria Senese [European Reference Network (ERN) for Rare Immunodeficiency, Autoinflammatory and Autoimmune Diseases (RITA) Center], Policlinico “Le Scotte”, Viale Bracci 16, 53100 Siena, Italy; 2 Clinical Paediatrics, Department of Molecular Medicine and Development, University of Siena, Siena, Italy; 3 Joint Academic Rheumatology Program, Medical School, National and Kapodistrian University of Athens, Athens, Greece; 4 Internal Medicine Department, Rheumatology and Clinical Immunology Unit, Faculty of Medicine, Cairo University, Giza, Egypt; 5 Faculty of Medicine, Newgiza University, 6th of October City, Egypt; 6 Rheumatology Unit, Department of Emergency and Organ Transplantation, University of Bari, Bari, Italy; 7 Rheumatology Unit, Department of Medicine, College of Medicine, King Saud University, Riyadh, Saudi Arabia; 8 College of Medicine Research Center, College of Medicine, King Saud University, Riyadh, Saudi Arabia; 9 Pediatric Rheumatology, Department of Pediatrics, College of Medicine, King Saud University, Riyadh, Saudi Arabia; 10 Rheumatology Division, Faculdade de Medicina, Hospital das Clinicas (HCFMUSP), Universidade de Sao Paulo, São Paulo, Brazil; 11 Pediatric Clinic, University of Brescia and Spedali Civili Di Brescia [European Reference Network (ERN) for Rare Immunodeficiency, Autoinflammatory and Autoimmune Diseases (RITA) Center], Brescia, Italy; 12 Rheumatology Unit, Department of Medical Sciences, University and AOU of Cagliari, Cagliari, Italy; 13 Rheumatology Unit, Azienda Ospedaliero-Universitaria Senese [European Reference Network (ERN) for Rare Immunodeficiency, Autoinflammatory and Autoimmune Diseases (RITA) Center], Siena, Italy; 14 University Department of Health Promotion, Mother and Child Care, Internal Medicine and Medical Specialties (PROMISE) “G. D’Alessandro”, University of Palermo, Palermo, Italy; 15 Rheumatology and Internal Medicine Department, Carol Davila University of Medicine and Pharmacy, Bucharest, Romania; 16 Department of Internal Medicine, Division of Rheumatology, Cerrahpasa Medical School, Istanbul University-Cerrahpasa, Istanbul, Turkey; 17 Behçet’s Disease Research Center, Istanbul University-Cerrahpasa, Istanbul, Turkey; 18 Division of Rheumatology, Ospedale Pediatrico Bambino Gesù, IRCCS [European Reference Network (ERN) for Rare Immunodeficiency, Autoinflammatory and Autoimmune Diseases (RITA) Center], Rome, Italy; 19 Department of Woman, Child and of General and Specialized Surgery, University of Campania “Luigi Vanvitelli”, Naples, Italy; 20 Division of Rheumatology, Department of Internal Medicine, Faculty of Medicine, Gazi University, Ankara, Turkey; 21 Department of Internal Medicine, Division of Rheumatology, Gazi University Hospital, Ankara, Turkey; 22 Department of Pediatrics, Pediatric Rheumatology Center, Giovanni XXIII Pediatric Hospital, University of Bari, Bari, Italy; 23 Unit of Pediatric Rheumatology, Azienda Socio-Sanitaria Territoriale (ASST) Gaetano Pini-Centro Specialistico Ortopedico Traumatologico (CTO), Milan, Italy; 24 Department of Ophthalmology and Otolaryngology, University of Bari, Bari, Italy; 25 Rheumatology Unit, Department of Biotechnological and Applied Clinical Sciences, University of L’Aquila, L’Aquila, Italy; 26 Rheumatology and Clinical Immunology, Department of Clinical and Experimental Sciences, University of Brescia and Spedali Civili [European Reference Network (ERN) for Rare Immunodeficiency, Autoinflammatory and Autoimmune Diseases (RITA) Center], Brescia, Italy; 27 Division of Gastroenterology, Ospedali Riuniti Villa Sofia-Vincenzo Cervello, Palermo, Italy; 28 Department of Precision Medicine, Università Degli Studi Della Campania Luigi Vanvitelli, Naples, Italy; 29 Department of Internal Medicine, Pulmonology, Allergy and Clinical Immunology, Central Clinical Hospital of the Ministry of National Defence, Military Institute of Medicine, Warsaw, Poland; 30 Internal Medicine and Rheumatology Department-St. Maria Hospital, Carol Davila University of Medicine and Pharmacy, Bucharest, Romania; 31 Division of Rheumatology, Department of Internal Medicine, Sivas Cumhuriyet University Medical Faculty, Sivas, Turkey; 32 Division of Rheumatology, Department of Internal Medicine, School of Medicine, Manisa Celal Bayar University, Manisa, Turkey; 33 Department of Immunology and Rheumatology, Instituto Nacional de Ciencias Médicas Y Nutrición Salvador Zubirán, Mexico City, Mexico; 34 Rheumatology and Immunology Unit, Internal Medicine Department, Mansoura University, Mansoura, Egypt; 35 Department of Autoimmune Diseases, Institut d’Investigacions Biomèdiques August Pi I Sunyer (IDIBAPS), Hospital Clínic of Barcelona [European Reference Network (ERN) for Rare Immunodeficiency, Autoinflammatory and Autoimmune Diseases (RITA) Center], University of Barcelona, Barcelona, Spain; 36 Pediatric Nephrology and Rheumatology Unit, Azienda Ospedaliero Universitaria (AOU) G Martino, Messina, Italy; 37 Pediatric and Neonatology Unit, Department of Maternal Infantile and Urological Sciences, Sapienza University of Rome, Polo Pontino, Latina, Italy; 38 Department of Medical Sciences, Rheumatology Unit, Azienda Ospedaliero-Universitaria S. Anna-Ferrara, University of Ferrara, Ferrara, Italy; 39 Department of Experimental and Clinical Medicine, University of Florence, Florence, Italy; 40 Centre for Inflammatory Diseases, Department of Medicine, Monash Medical Centre, Monash University, Clayton, Victoria Australia; 41 Ophthalmology Unit, Department of Medicine, Surgery and Neurosciences, University of Siena and Azienda Ospedaliero-Universitaria Senese [European Reference Network (ERN) for Rare Immunodeficiency, Autoinflammatory and Autoimmune Diseases (RITA) Center], Siena, Italy; 42 Bioengineering and Biomedical Data Science Lab, Department of Medical Biotechnologies, University of Siena, Siena, Italy; 43 Department of Life Sciences and Global Health, Fondazione Policlinico Universitario A. Gemelli IRCCS, Rome, Italy; 44 Rare Diseases and Periodic Fevers Research Centre, Università Cattolica del Sacro Cuore, Rome, Italy

**Keywords:** Behçet’s syndrome, Arthritis, Pediatric rheumatology, International registry, Rare diseases

## Abstract

This study aims to describe musculoskeletal manifestations (MSM) in children with Behçet’s syndrome (BS), their association with other disease manifestations, response to therapy, and long-term prognosis. Data were retrieved from the AIDA Network Behçet’s Syndrome Registry. Out of a total of 141 patients with juvenile BS, 37 had MSM at disease onset (26.2%). The median age at onset was 10.0 years (IQR 7.7). The median follow-up duration was 21.8 years (IQR 23.3). Recurrent oral (100%) and genital ulcers (67.6%) and pseudofolliculitis (56.8%) were the most common symptoms associated with MSM. At disease onset, 31 subjects had arthritis (83.8%), 33 arthralgia (89.2%), and 14 myalgia (37.8%). Arthritis was monoarticular in 9/31 cases (29%), oligoarticular in 10 (32.3%), polyarticular in 5 (16.1%), axial in 7 (22.6%). Over time, arthritis became chronic-recurrent in 67.7% of cases and 7/31 patients had joint erosions (22.6%). The median Behçet's Syndrome Overall Damage Index was 0 (range 0–4). Colchicine was inefficacious for MSM in 4/14 cases (28.6%), independently from the type of MSM (*p* = 0.46) or the concomitant therapy (*p* = 0.30 for cDMARDs, *p* = 1.00 for glucocorticoids); cDMARDs and bDMARDs were inefficacious for MSM in 6/19 (31.4%) and 5/12 (41.7%) cases. The presence of myalgia was associated with bDMARDs inefficacy (*p* = 0.014). To conclude, MSM in children with BS are frequently associated with recurrent ulcers and pseudofolliculitis. Arthritis is mostly mono- or oligoarticular, but sacroiliitis is not unusual. Prognosis of this subset of BS is overall favorable, though the presence of myalgia negatively affects response to biologic therapies. ClinicalTrials.gov Identifier: NCT05200715 (registered on December 18, 2021).

## Introduction

Behçet’s syndrome (BS) is a rare inflammatory disorder currently framed at the border of autoinflammation and autoimmunity. Emerging clinical, experimental, genetic and therapeutic data place this nosological entity in the immunological disease continuum of MHC-I-opathies with psoriasis, psoriatic arthritis, and spondyloarthropathies [1]. These diseases share a common pathophysiologic ground and the predilection for environmental barrier surfaces (mucosae, gastro-intestinal tract, skin) and for tissues susceptible to mechano-inflammation such as entheses, including anchorage sites in the eye, vessel walls and valve regions. The heterogeneous clinical expression of BS has been extensively described and multiple data converge on the definition of phenotype clusters, among which, the papulopustular-articular cluster, is variably associated with mucosal ulcers, and largely preserved across all studies [2]. As for the pediatric age, the articular cluster ranked second in frequency in a cluster analysis recently performed by the PeRA research group, accounting for 15.6% of juvenile BS patients [3]. Articular symptoms are reported by 30.9–42.9% of children in the largest pediatric cohorts, with some regional variability (13% in a Chinese cohort, 63% in a Turkish one) [4–11]. Moreover, they are frequently observed at disease onset [10]. These studies largely report mono- or oligoarthritis with preference for large joints and enthesitis [10, 12, 13], while axial spondyloarthritis seems less common [14]; joint manifestations are rarely destructive and mostly self-limiting [15]. However, an exhaustive description of musculoskeletal manifestations (MSM) in children with BS is lacking, as data are mostly derived from cohorts of adult patients. This present study aims to characterize MSM in children with BS at disease onset and during the disease course, highlighting the frequency of co-existing clinical manifestations, the overall response of MSM to different treatment regimens, and the long-term prognosis of this subset of patients.

## Methods

### Data collection

This is a registry-based observational multicenter study. Data were collected in the AIDA Network Behçet’s Syndrome Registry by 13 international reference centers for BS. The diagnosis of BS should be made according to the International Study Group (ISG) or International Criteria for Behçet’s Disease (ICBD) or PEDiatric Behçet’s Disease (PEDBD) criteria [10, 16, 17]. For the purpose of this study, we extracted the records of patients fulfilling the following entry criteria: (i) onset of BS before 18 years of age, and (ii) presence of MSM at disease onset (arthritis, arthralgia, myositis and/or myalgia).

Demographic, clinical, clinimetric, and therapeutic data of each patient were collected retrospectively from the clinical charts and prospectively during routine follow-up visits by the treating physician. Data were collected at the time of disease onset, at the start of treatment with colchicine, conventional (c-) and biologic (b-) disease-modifying anti-rheumatic drugs (DMARDs), at 3-, 6- and 12 months and at the last assessment during treatment. Only treatment courses started when the patient had active MSM were analyzed. The information on the response of MSM to a specific drug was retrieved from the registry based on the answer to the following question: “The Investigator should specify which disease manifestation(s) persisted despite … treatment”. Further information on MSM (pattern of arthritis, affected joints, disease activity, disease-related damage, presence of autoantibodies) was collected at disease onset and at each prospective visit, while retrospective forms dedicated to past treatments included the assessment of active/inactive MSM at 0, 3, 6, 12 months and at the last visit. Disease-related damage was measured using the total Behçet’s Syndrome Overall Damage Index (BODI), specific BODI musculoskeletal items (osteoporotic fracture or vertebral collapse, muscle atrophy and bone avascular necrosis) and the presence of joint erosions at the end of follow-up [18].

### Statistical analysis

Statistical analysis was performed using JASP open-source statistics package version 0.16.3. Descriptive statistics included sample sizes, mean and standard deviation or median and interquartile range (IQR), as appropriate. Shapiro–Wilk test was used to assess normality distribution of data. Associations between categorical variables (i.e., association between different symptoms and response to treatment) were analyzed using contingency tables with Chi-Square test with Yates' continuity correction. Differences between paired categorical variables (i.e., presence/absence of MSM at different timepoints during treatment) were assessed using McNemar’s test. Differences between paired continuous variables (i.e., prednisone load at different timepoints during treatment) were analyzed by Friedman’s test and Conover’s post-hoc test. Dichotomic outcomes were predicted by logistic regression analysis. The threshold for statistical significance was set to *p* < 0.05 and all *p-*values were two-sided.

### Ethical considerations

The study protocol conformed to the tenets of the Declaration of Helsinki and was approved by the local Ethics Committee of the University of Siena (Reference No. 14951). Written informed consent for using clinical data for research purposes was obtained according to the local institutional review board guidelines.

## Results

### Demographic data

By data lock in June 2022, 722 subjects were enrolled in the AIDA Network Behçet’s Syndrome Registry, among whom 141 had disease onset in the pediatric age (19.5%). Thirty-seven children (26.2% of the pediatric group) showed MSM upon disease onset. The demographic and classificative information of the cohort is displayed in Table 1.

**Table 1:** Demographic data and clinical features at disease onset in pediatric Behçet’s syndrome patients with musculoskeletal symptoms [10, 16, 17]

Children with MSM at BS onset	*N* = 37	26.2% of the pediatric cohort
Female:male	23:14	
Ethnic origin	31 Caucasian (83.8%), 3 Arab (8.1%), 1 Romanian (2.7%), 1 Mulatto (2.7%), 1 missing (2.7%)	
Median age at disease onset	10.0 years (IQR 7.7)	Range 1.0–17.8
Median diagnostic delay	14.2 years (IQR 20.9)	Range 0–60.1
Median disease duration at the last follow-up	21.8 years (IQR 23.3)	Range 1.4–64.7
Familial history of Behçet’s syndrome	6/37	16.2%
Classification of patients	ISG criteria	30/37 (81.1%)
	ICBD criteria	33/37 (89.1%)
	PEDBD criteria	30/37 (81.1%)

Clinical manifestations	N. of patients	% of patients
Recurrent oral aphthosis	37	100%
Skin manifestations	28	75.7%
Erythema nodosum	10	27%
Pseudofolliculitis	21	56.8%
Skin ulcers	2	5.4%
Suppurative panniculitis	1	2.7%
Recurrent genital aphthosis	25	67.6%
Fever of unknown origin	17	45.9%
Gastrointestinal involvement	13	35.1%
Abdominal pain/ nausea/ diarrhoea	12	32.4%
Typical aphthae seen by endoscopy	2	5.4%
Macroscopic/microscopic inflammatory lesions seen by endoscopy	6	16.2%
Endoscopy not done	5	13.5%
Negative endoscopy	2	5.4%
Ocular involvement	13	35.1%
Neurologic involvement	10	27%
CNS manifestations	7	18.9%
PNS manifestations	5	13.5%
Psychiatric symptoms	3	8.1%
Vascular involvement	7	18.9%
Cardiac involvement	2	5.4%
Arrhythmia	1	2.7%
Myopericarditis	1	2.7%
Intracardiac thrombosis	1	2.7%
Lymphadenopathy	5	13.5%
Hepatomegaly	1	2.7%
Pleuritis	1	2.7%
Orchitis	1	2.7%

*BS* Behçet’s syndrome; *CNS* central nervous system; *ICBD* International Criteria for Behçet’s Disease; *ISG* international study group; *MSM* musculoskeletal manifestations; *PEDBD* PEDiatric Behçet’s disease; *PNS* peripheral nervous system

### Clinical features of Behçet’s syndrome at disease onset

**Fig. 1:**
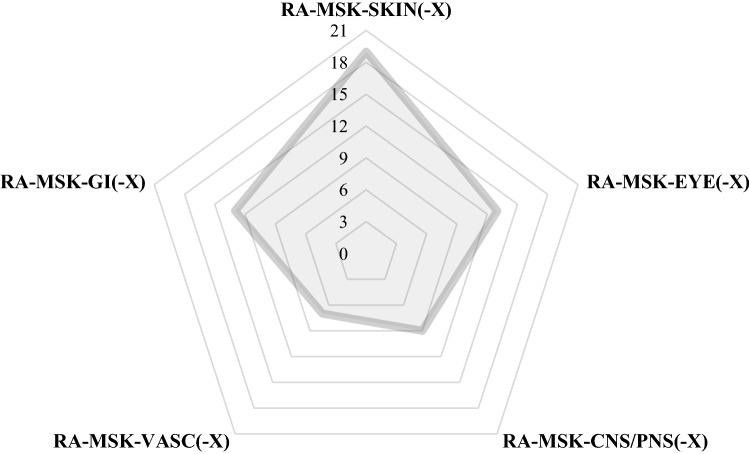
Diagram showing the distribution of symptom associations in the pediatric musculoskeletal Behçet’s syndrome cohort. *CNS* Central Nervous System manifestations; *EYE* ocular manifestations; *GI* gastrointestinal manifestations; *MSK* musculoskeletal manifestations; *PNS* Peripheral Nervous System manifestation; *RA* recurrent oral and/or genital aphthosis; *SKIN* cutaneous manifestations; *VASC* vascular manifestations; “X” indicates the variable association of further disease manifestations

At disease onset, recurrent oral ulceration was observed in 37/37 patients (100%), recurrent genital ulceration in 25/37 (67.6%), and skin manifestations in 28/37 (75.7%). Further clinical manifestations observed in association with MSM are described in Table 1. The Human Leukocyte Antigen (HLA) B51 haplotype was detected in 19/37 patients (51.4%). Ten out of 37 subjects (27%) had only muco-cutaneous manifestations and MSM at disease onset. Figure 1 shows the distribution of symptom associations in the whole cohort.

### Characterization of MSM at disease onset and over time

At disease onset, 31/37 subjects had arthritis (83.8%), 33/37 arthralgia (89.2%), and 14/37 myalgia (37.8%). Arthritis was monoarticular in 9/31 cases (29%), oligoarticular in 10/31 (32.3%), polyarticular in 5/31 (16.1%), axial in 7/31 (22.6%). Arthritis involved the sacroiliac joints in 7/31 patients (22.6%), large joints (ankle, wrist, elbow, knee) in 6/31 patients (19%), small joints of hands and feet in 1 patient (3.2%), while this information was missing in 17 cases (54.8%). Arthritis was erosive in 6/31 children (19.4%), non-erosive in 22 (71%), while the information is missing in 3 cases (9.7%). Five children out of 31 (16.1%) were positive for anti-nuclear antibodies. The HLA B27 haplotype was absent in all patients tested (18/37, 48.6%). Among the 14 subjects with myalgia, 3 had deep or superficial venous thrombosis, 3 erythema nodosum, 1 sensory polyneuropathy, 1 multiple sclerosis-like neurological manifestations, 6 symptoms suggestive of superimposed fibromyalgia (fatigue, sleep disorder, tensive headache, dizziness, memory loss, tremor) not otherwise explained.

Follow-up data were available in the registry for 28/37 patients (75.7%). At the last follow-up visit (median disease duration of 21.8 years), 16 patients out of 28 had active MSM: arthralgia was present in 10/28 (35.7%), arthritis in 9/28 (32.1%), and myalgia in 7/28 (25.0%). Among the 31 cases with arthritis at disease-onset, the symptom resolved in 6 over time (19.4%) and 21 became chronic-recurrent (67.7%), while this information is missing in 4 patients. The course of arthritis was progressive in 6 cases: 2 children with monoarthritis developed oligoarthritis over time, whereas in 4 cases the axial skeleton was also involved late in the disease course. At the end of follow-up (mean age 29.0 ± 14.9 years [range 10.0 – 64.1]), 7/31 patients had erosive manifestations in the affected joints (22.6%), 1 patient had muscle atrophy, and the median total BODI was 0 (IQR 1.7, range 0–4). No predictive factors of the development of chronic erosive arthritis were identified.

### Treatment courses

We analyzed 14 treatment courses with colchicine, 19 with cDMARDs, and 12 with bDMARDs in patients with active MSM.

**Fig. 2:**
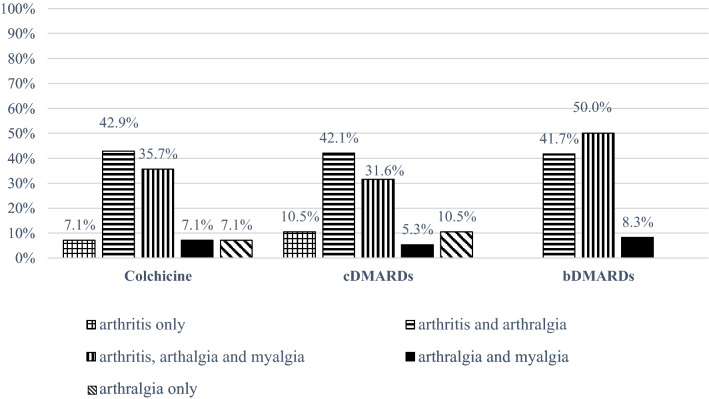
Distribution of active musculoskeletal manifestations at baseline across the treatment groups. *bDMARDs* biologic disease-modifying antirheumatic drugs; *cDMARDs* conventional disease-modifying antirheumatic drugs

The treatment course details are provided in Table 2, while the distribution of active MSM at baseline among the treatment groups is displayed in Fig. 2.

**Table 2:** Description of treatment courses in patients with active musculoskeletal manifestations

Therapy	Treatment courses	
Colchicine	*N* = 14	10 monotherapy 1 AZA 1 MTX 2 SSZ
	Median treatment duration = 16 months (IQR 36, range 2 – 75)	
cDMARDs	N = 19 7 AZA 5 SSZ 4 MTX 2 HCQ 1 CsA	12 monotherapy 7 colchicine
	Median treatment duration = 12 months (IQR 33, range 1 – 60)	
bDMARDs	N = 12 6 ADA 1 ETN 2 IFX 1 ANA 2 CAN	3 monotherapy 7 colchicine 2 cDMARDs
	Mean treatment duration = 23.4 ± 18.1 months (range 3 – 60)	

*ADA* adalimumab; *ANA* anakinra; *AZA* azathioprine; *bDMARDs* biologic disease-modifying anti-rheumatic drugs; *CAN* canakinumab; *cDMARDs* conventional disease-modifying anti-rheumatic drugs; *CsA* cyclosporin A; *ETN* etanercept; *HCQ* hydroxychloroquine; *IFX* infliximab; *IQR* interquartile range; *MTX* methotrexate; *SSZ* sulfasalazine

The MSM were nonresponsive to colchicine in 4/14 cases (28.6%). There was no significant association between the different types of musculoskeletal expression and colchicine efficacy (*p* = 0.46). Also, colchicine efficacy was not associated with the concomitant cDMARD or corticosteroid therapy at baseline (*p* = 0.30 and *p* = 1.00, respectively). Colchicine was discontinued in 2 patients because of lack of efficacy, whereas it is ongoing in 11 patients as monotherapy or in association with other drugs (information is missing in 1 patient).

The MSM were nonresponsive to cDMARDs in 6/19 cases (31.4%). There was no significant association between the different types of MSM and cDMARDs efficacy (*p* = 0.33). While the concomitant use of corticosteroids and colchicine did not significantly influence cDMARD efficacy (*p* = 0.67 and *p* = 0.062, respectively), a tendency was observed in favor of the co-administration of colchicine (efficacy in 6/6 cases) *versus* cDMARD monotherapy (efficacy in 4/10 cases). At the last follow-up visit, cDMARDs had been discontinued in 10 cases because of lack of efficacy (3/10), loss of efficacy (1/10), and adverse events (4/10), whereas 5 cases were still on cDMARDs (data missing for 4 patients).

The musculoskeletal involvement was considered nonresponsive to bDMARDs in 5/12 cases (41.7%). There was a significant association between the different types of MSM and bDMARDs efficacy (*p* = 0.014) with a tendency towards a lesser efficacy in patients with myalgia than without (*p* = 0.06). The bDMARD efficacy was not associated with the concomitant treatment, and in particular with the concomitant use of colchicine/cDMARDs or corticosteroids (*p* = 0.93, *p* = 1.00). At the last follow-up visit, bDMARDs had been discontinued in 8 cases because of lack of efficacy (4/8), loss of efficacy (1/8), adverse events (1/8), pregnancy (1/8), poor compliance (1/8) and non-medical reasons (1/8), whereas they were still given in 3 cases.

**Fig. 3:**
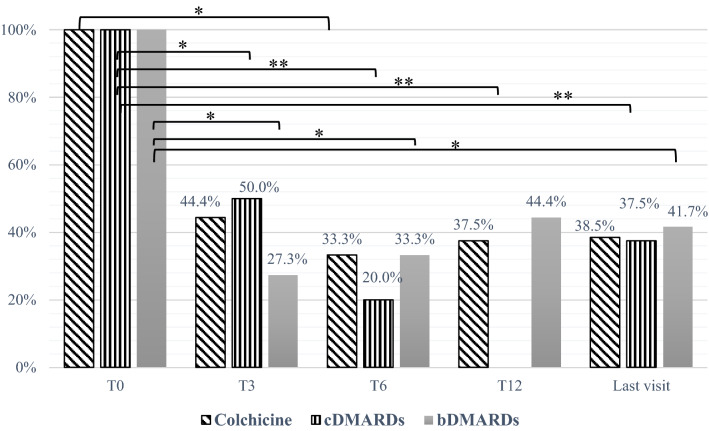
Valid percentages of patients showing persistent musculoskeletal manifestations at 3, 6, 12 months of treatment and at the last follow-up visit while on colchicine, cDMARDs and bDMARDs. *bDMARDs* biologic disease-modifying antirheumatic drugs; *cDMARDs* conventional disease-modifying antirheumatic drugs. *p < 0.05; **p < 0.01

**Fig. 4:**
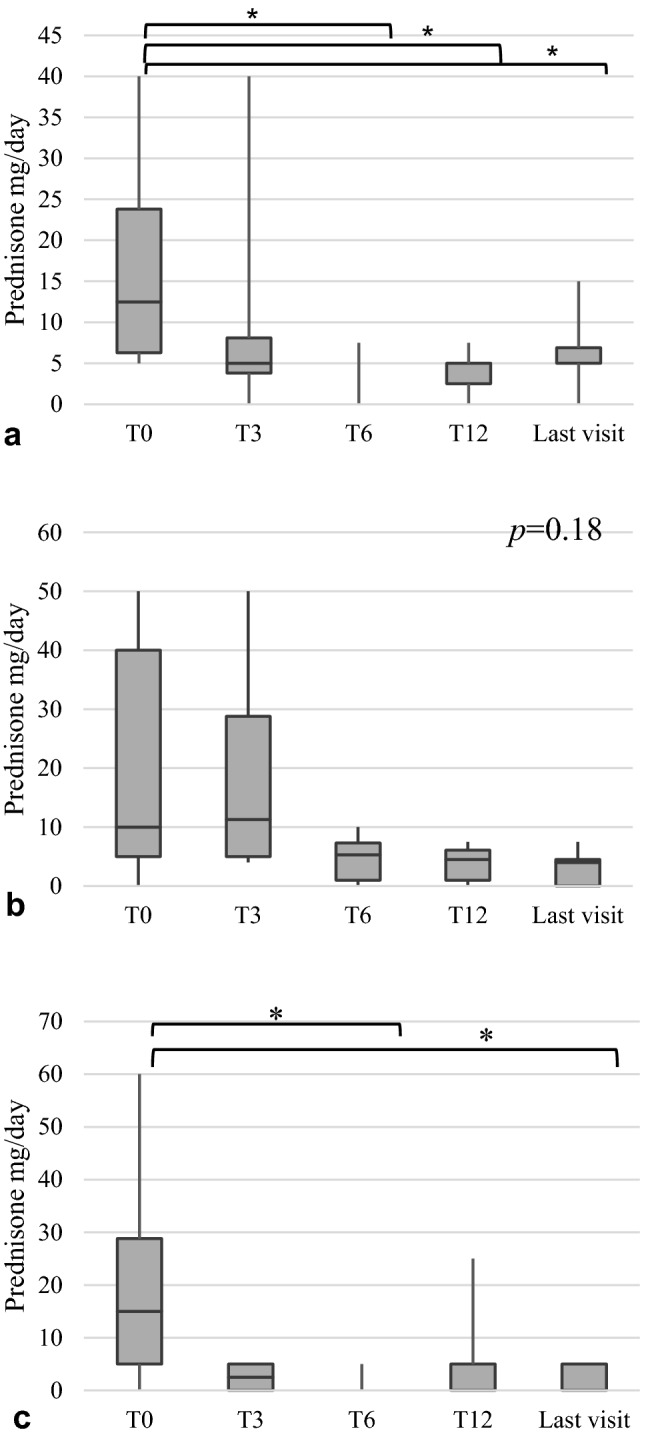
Reduction of prednisone load over time in patients with active musculoskeletal manifestations treated with colchicine (**5a**), cDMARDs (**5b**) and bDMARDs (**5c**). *p < 0.05

The percentage of patients showing persistent MSM at 3, 6, 12 months of treatment and at the last follow-up visit, while on colchicine, cDMARDs and bDMARDs, is shown in Fig. 3. Figure 4a–c shows the reduction of prednisone load over time in each treatment group.

## Discussion

This study provides a general overview of a subset of patients with childhood-onset BS presenting with non-specific MSM: these children may seek medical attention from the general pediatric or pediatric rheumatology services because of arthralgia, myalgia or joint swelling and will be evaluated upon the suspicion of rheumatologic diseases occurring in childhood. In particular, they represent one-fourth of the whole pediatric BS cohort from the AIDA Network Behçet’s Syndrome Registry. This percentage seems lower than expected according to the medical literature, but it should be considered that we included only patients with MSM at disease onset to evaluate the musculoskeletal symptoms during the whole disease course. In our cohort, the frequency of females was almost double the one of males, and median age at onset was around 10 years, not differing from that described in the whole pediatric BS population in previous studies [5, 9, 19]. Also, the mean age of 10.02 ± 3.79 years at the onset of articular symptoms is consistent with that observed in one of the major cohorts of pediatric BS in Europe [10]. BS classification criteria are useful tools supporting the diagnostic process, and the ICBD ones may be preferably used in children at disease onset by virtue of their higher sensitivity (around 90% in this cohort), although with caution in areas where BS prevalence is very low [20]. Also, the familial history may provide key clues to the diagnosis in about 16% of cases, and should go through all cardinal features of BS, especially the presence of arthritis, enthesitis and papulopustular lesions, since they belong to the same phenotypic cluster highly preserved in familial BS [21].

The frequency of BS manifestations associated with MSM in our cohort supports the hypothesis of a distinct articular-papulopustular cluster, in agreement with cases observed in the adult setting [21–23]. Indeed, we found that up to 1/3 of children with MSM at onset had only mucocutaneous manifestations as concomitant symptoms, specifically recurrent oral aphthosis (100%), recurrent genital ulcers (67.6%), and pseudofolliculitis (56.8%). The frequency of concomitant pseudofolliculitis was found higher than we expected based on previously published data from an Italian cohort of juvenile-onset BS, where pseudofollicular/papulopustular lesions were observed in only 21.9% of patients; interestingly, in that cohort, musculoskeletal disease was reported at disease onset only in 12.5% of cases [19]. When considering the medical literature about adult BS, pseudofolliculitis was the most common manifestation during arthritic attacks in a prospective study from Iran [24]; moreover, the association between articular and cutaneous features of BS has been also extensively studied in adults by a research group from Turkey at the beginning of the century: they nicely demonstrated that acne lesions and arthritis are likely to occur together and remain clustered also when included in factor analysis [21, 23, 25]. On the other hand, according to the cluster analysis recently performed by the PeRA research group, arthritis accounted for a specific cluster (C2) in juvenile BS, and in this cluster papulopustular lesions were reported in 37.1% of cases and enthesitis in 62.9% [3]. In this context, the results of our study should be considered carefully since children with musculoskeletal symptoms appearing later in the disease course might have been excluded because they failed to meet the inclusion criteria. In fact, different BS manifestations may appear with a considerable latency, which also accounts for a diagnostic delay in pediatric BS (median 14.2 years in this cohort) [26].

According to our results, children with BS are likely to show up with both arthralgia and arthritis, the latter being mono- or oligoarticular in almost 2/3 of cases. Large joints (ankle, wrist, elbow, knee) have been most frequently involved at onset in childhood, although precise information on the site and laterality of active arthritis at the different timepoints were sparsely available in the retrospective aspects of the registry. Gallizzi et al. observed mono- or oligoarthritis in 68% of a cohort of 110 Italian children with BS during the whole disease course [5]. Notably, mono- or oligoarthritis accounted for over 90% of arthritis patterns in a broad cohort of adults from Iran; the same research group found knees, ankles and wrists, followed by small joints of hands and feet, as the most frequently involved sites of arthritis, mostly with an asymmetrical pattern [14, 24]. With this regard, the analysis of data from the prospective section of the AIDA Network Behçet’s Syndrome Registry may provide in the future conclusive results also in the pediatric context.

The occurrence of spondyloarthritis in BS deserves separate consideration. The Turkish colleagues observed a higher ultrasound enthesopathy score in adult BS patients with acne and arthritis compared to other BS patients as well as patients with rheumatoid arthritis and healthy controls; on the other hand, they found neither radiographic sacroiliitis, nor HLA B27 association in this BS cluster, questioning the hypothesis that BS belongs to the spondyloarthropathy group [13, 27, 28]. This is not the case in the present study, as sacroiliac inflammation was detected in 7/37 subjects at disease onset and appeared later in the clinical history of 4 additional patients, resulting in axial involvement in almost 1/3 of the cohort, although HLA B27 was absent in all cases. Indeed, the incidence of sacroiliitis in BS has been a matter of historical debate, also because of inhomogeneous classification criteria and imaging methods [29–31]. In particular, it has to be mentioned that most old studies addressing this issue in adults relied on the pelvis antero-posterior radiograph, whose interpretation may be biased by a high intra-observer variation and not consistent with more accurate methods, such as computed tomography (CT) and Tc-99 m-MDP bone scintigraphy [31–33]. With this respect, Sahin et al. detected sacroiliac joint inflammation by Tc-99 m-MDP bone scintigraphy in 25% of BS patients who were clinically asymptomatic and had normal pelvis radiography [31]. However, pelvis X-ray is not suitable for the diagnosis of sacroiliitis in the pediatric age, since typical changes may not be seen for many years after the onset of inflammation, while pelvis CT should be avoided to limit radiation exposure. Hence, results from this pediatric study, largely based on the use of magnetic resonance imaging (MRI), may represent a novel finding in this field, not directly comparable with the previous literature data and worth further consideration in our research agenda.

According to this long-term follow-up study, arthritis tends to resolve over time in 20% of children with BS, while most of them have recurrent disease. In a few cases further joints may be involved over time (peripheral joints or the axial skeleton). Moreover, at the end of follow-up, 23% of patients from this cohort had erosive manifestations in the affected joints, which is quite surprising given the usually mild course of arthritis described in the adult population. Peripheral destructive arthritis associated with BS has been anecdotally reported, with rheumatoid-like or psoriatic arthritis-like features [34, 35]. However, the significance of this finding in our cohort should be mitigated since we were unable to clarify the severity of erosive manifestations in each patient and, moreover, the applied radiological technique was not known, which is crucial for drawing sharp conclusions on this issue. Indeed, the median total BODI score at the end of follow-up was 0, and none of the participants scored positive in the musculoskeletal items of the BODI, except for one patient showing muscle atrophy in adulthood, possibly as a consequence of multiple comorbidities (diabetes, peripheral neuropathy, neoplasm).

The benign prognosis of this subset of patients is also supported by the high efficacy of colchicine for MSM, independently of the type of clinical manifestations (arthralgia, arthritis and/or myalgia) or concomitant treatment with cDMARDs or corticosteroids. Indeed, colchicine is recommended as first-line therapy for BS with MSM in the most recent international treatment recommendations [36, 37]. On the other hand, MSM were not responsive to biologic agents in 42% of patients, with a tendency towards a lesser efficacy in patients with myalgia than those without. Myalgia in subjects with BS can imply several causes, including focal vasculitic myositis, myo-fasciitis, thrombophlebitis, peripheral neuropathy, erythema nodosum, drug-induced neuromyopathy, and superimposed fibromyalgia. The latter is supposed to be the major cause of myalgia in our cohort (43%), followed by deep or superficial venous thrombosis, erythema nodosum and neurological involvement. Interestingly, it was previously observed by Ayar et al. that both BS activity and its perception by patients and clinicians are higher in female patients with fibromyalgia than those without, even without considering arthralgia among symptoms concurring to disease activity. Moreover, in the same study, the presence of fibromyalgia was associated with the presence of active genital ulcers [38]. Given the prevalent female representation (62%) and the high frequency of genital ulcers along with MSM (68%) in our cohort, previous data agree with the present study.

In conclusion, this study provides a comprehensive overview of the natural history of MSM of BS with onset during childhood. Better characterizing minor features of the syndrome at onset would help in the diagnostic work-up of pediatric patients, in whom BS is the cause of nonspecific musculoskeletal complaints, which should be promptly recognized to avoid diagnostic delay and prevent major organ complications. One of the main strengths of this study is that data derive from a clinical registry including pediatric and adult patients, which allowed us to evaluate the disease evolution in the long term. Also, considering the low prevalence of juvenile BS, the numerosity of this cohort is quite high. On the other hand, retrieving data from both retrospective and prospective instruments of the registry may have resulted in inhomogeneous information. Moreover, we enrolled in this study only patients with MSM at disease onset aiming to assess the evolution of MSM during the whole clinical history; this decision turned out to be also a limitation in interpreting the results, since patients showing MSM later during the disease course have been excluded. With these considerations in mind, our study does not break away from what has been described in largest adult cohorts with regard to the articular expression in BS and its favorable prognosis. Based on our results, however, we suggest to keep a high clinical suspicion for sacroiliitis, which should be ruled out by pelvis MRI with specific sequences for sacroiliac joints interpreted by an experienced pediatric radiologist. Moreover, as low quality of life, depression and pathological anxiety levels in BS are often observed in mutual connection with disease activity also during the pediatric age, thus, a close collaboration among pediatricians, rheumatologists, physiatrists, and psychiatrists is highly recommended for the optimal therapeutic management of such complex patients.
